# The Mediation Role of Dynamic Multisensory Processing Using Molecular Genetic Data in Dyslexia

**DOI:** 10.3390/brainsci10120993

**Published:** 2020-12-16

**Authors:** Sara Mascheretti, Valentina Riva, Bei Feng, Vittoria Trezzi, Chiara Andreola, Roberto Giorda, Marco Villa, Ginette Dionne, Simone Gori, Cecilia Marino, Andrea Facoetti

**Affiliations:** 1 Child Psychopathology Unit, Scientific Institute, IRCCS E. Medea, 23842 Bosisio Parini, Italy; sara.mascheretti@lanostrafamiglia.it (S.M.); valentina.riva@lanostrafamiglia.it (V.R.); vittoriatrezzi@gmail.com (V.T.); chiara.andreola@etu.parisdescartes.fr (C.A.); 2 École de Psychologie, Laval University, Québec, QC G1V 0A6, Canada; Bei.Feng@psy.ulaval.ca (B.F.); Ginette.Dionne@psy.ulaval.ca (G.D.); 3 Laboratoire de Psychologie du Développement et de l’Éducation de l’Enfant (LaPsyDÉ), Universitè de Paris, 75005 Paris, France; 4 Molecular Biology Laboratory, Scientific Institute, IRCCS E. Medea, 23842 Bosisio Parini, Italy; roberto.giorda@lanostrafamiglia.it (R.G.); marco.villa@lanostrafamiglia.it (M.V.); 5 Department of Human and Social Sciences, University of Bergamo, 24100 Bergamo, Italy; simone.gori@unibg.it; 6 Department of Psychiatry, University of Toronto, Toronto, ON M5T 1R8, Canada; 7 The Division of Child and Youth Psychiatry, Centre for Addiction and Mental Health (CAMH), Toronto, ON M6J 1H4, Canada; 8 Developmental Cognitive Neuroscience Lab, Department of General Psychology, University of Padua, 35131 Padua, Italy

**Keywords:** candidate genes, developmental dyslexia, endophenotypes, mediation, multisensory temporal processing

## Abstract

Although substantial heritability has been reported and candidate genes have been identified, we are far from understanding the etiopathogenetic pathways underlying developmental dyslexia (DD). Reading-related endophenotypes (EPs) have been established. Until now it was unknown whether they mediated the pathway from gene to reading (dis)ability. Thus, in a sample of 223 siblings from nuclear families with DD and 79 unrelated typical readers, we tested four EPs (i.e., rapid auditory processing, rapid automatized naming, multisensory nonspatial attention and visual motion processing) and 20 markers spanning five DD-candidate genes (i.e., *DYX1C1*, *DCDC2*, *KIAA0319*, *ROBO1* and *GRIN2B*) using a multiple-predictor/multiple-mediator framework. Our results show that rapid auditory and visual motion processing are mediators in the pathway from *ROBO1*-rs9853895 to reading. Specifically, the T/T genotype group predicts impairments in rapid auditory and visual motion processing which, in turn, predict poorer reading skills. Our results suggest that *ROBO1* is related to reading via multisensory temporal processing. These findings support the use of EPs as an effective approach to disentangling the complex pathways between candidate genes and behavior.

## 1. Introduction

Developmental dyslexia (DD) is a complex heritable neurodevelopmental disorder characterized by impaired reading acquisition, in spite of adequate neurological and sensorial functioning, educational opportunities and average intelligence [1]. DD is one of the most common neurodevelopmental disorders affecting about 7% of school-age children across languages and is often associated with undesirable outcomes [2], as well as negative social impact and economic burden [3].

Subsequent to earlier descriptions of high familial aggregation of DD [4], substantial heritability typical of a complex trait has been reported [5]. Although they have not been found to be associated with DD-related traits by recent GWAS [6,7,8,9] and in a large cross-linguistic sample [10], nine genes have been replicated in at least one independent sample by candidate genes studies: *DYX1C1*, *DCDC2*, *KIAA0319*, *C2orf3*, *MRPL19*, *ROBO1*, *FAM176A*, *NRSN1*, *KIAA0319L* and *FMR1* [11]. In our previous studies, we reported the association of single nucleotide polymorphisms (SNPs) spanning the *DYX1C1*, *DCDC2*, *KIAA0319*, *ROBO1*, and *GRIN2B* genes with DD and DD-related quantitative traits in Italian nuclear families with DD [12,13,14,15,16,17,18]. Recent evidence has shown that *DYX1C1*, *DCDC2*, *KIAA0319*, *ROBO1*, and *GRIN2B*, affect neuronal migration, neurite outgrowth, cortical morphogenesis and ciliary structure and function. On the contrary, little is known about the *C2orf3* and *MRPL19* candidate genes whose expression is strongly correlated with *DYX1C1*, *ROBO1*, *DCDC2* and *KIAA0319* across different brain regions [11].

Although genetic results have contributed to the understanding of the molecular mechanisms involved at an etiological level, pathways linking genetic variations to clinical manifestation remain poorly understood. Testing endophenotypes (EPs) or intermediate phenotypes (IPs) as mediating variables has been proposed as a useful approach to disentangling the complex pathways between genes and behavior [19,20,21,22]. Furthermore, testing the mediating effects of EPs/IPs is particularly relevant in candidate gene studies of complex disorders, as it can improve the understanding of clinical heterogeneity and, conceivably, help reshape the classical nosological systems and diagnostic categories and pave the way for targeted remediation treatments [19,20,21,22]. EPs/IPs reflect lower-level neurophysiological, biochemical, endocrinological, neuroanatomical, cognitive or neuropsychological processes [19,23,24] associated with a trait or disorder and might link specific genes to a phenotype [25,26].

Some well-studied cognitive (i.e., phonological awareness—PA, rapid automatized naming—RAN, visual and auditory attention) and sensory mechanisms (i.e., rapid auditory processing—RAP, visual motion processing) have been associated with and predict DD [27,28,29,30,31,32,33,34,35,36,37,38,39,40,41,42,43,44,45,46,47,48,49,50]. Among the above-cited IPs, visual motion processing, RAP, multisensory non-spatial attention and RAN have recently been established as solid and valuable EPs for DD [51]. Heritability was found to be high for all these traits [51,52,53,54,55,56,57]. Moreover, recent findings have shown associations between DD-candidate risk genes and visual motion processing and RAN. A deletion in intron 2 of the *DCDC2* gene has been specifically associated with a visual motion deficit underlying the magnocellular-dorsal (M-D) stream in both subjects with DD and typical readers [58,59]. In a Canadian sample with DD, the *DYX1C1*-rs3743205 showed significant association with RAN [60].

Several animal studies have tested the links between DD-candidate the genes and cognitive and sensorial processes underlying reading acquisition. Although negative findings have also been reported [61], in utero RNAi of *DYX1C1* has been associated with deficits in RAP, spatial working memory performance, learning and memory performance [62,63]. The embryonic RNAi of *Kiaa0319* expression has resulted in RAP and spatial learning deficits [64]. *Dcdc2a* knockout mice have shown deficits in visuospatial memory, visual discrimination and long-term memory, working memory, reference memory and auditory processing [65,66], as well as increased excitability and decreased temporal precision in action potential firing [67], and increased functional excitatory connectivity between layer 4 lateral connections in the somatosensory neocortex mediated by subunit *Grin2B* [68].

While the above findings provide initial evidence that specific links between molecular genetic variants and EPs/IPs exist, evidence in support of a mediating role of EPs/IPs in the pathway from genes to DD is missing. In this study, we conducted a mediation analysis to concurrently test direct and indirect effects from multiple predictors (i.e., 20 SNPs spanning five DD-candidate genes) to DD via multiple mediators (i.e., visual motion processing, RAP, multisensory non-spatial attention and RAN) in a sample of 223 siblings from nuclear families with DD and 79 unrelated typical readers. Using multiple predictors yields an estimate of the unique effect of each SNP upon the behavioral phenotype (directly and indirectly through the mediator), relative to the other polymorphisms in the model [69]. In addition, using multiple mediators allows researchers to: (i) determine whether the set of mediators mediates the effect of genes on the behavioral outcomes; (ii) explore the extent to which specific EPs account for the association between genotype and phenotype, after having accounted for the presence of other mediators in the model; (iii) reduce the likelihood of parameter bias due to omitted variables; and (iv) pit competing theories against one another within a single model [70]. By concurrently testing direct and indirect effects from multiple SNPs spanning historical DD-candidate genes to reading (dis)ability via multiple mediators, the present study aimed to represent a step forward from our previous analyses. Based on our own previous findings and the literature, we hypothesized that SNPs spanning historical DD-candidate genes would be associated with decreased reading performance via their impact on the cognitive and sensorial EPs that support and predict reading skills.

## 2. Materials and Methods

The study was conducted in accordance with the Declaration of Helsinki and the protocol was approved by the Scientific Review Board and the Bioethics Committee of the Scientific Institute, IRCCS Eugenio Medea (Ricerca Corrente “2019, 2020”).

### 2.1. Sample

The sample consisted of two merged subsamples. The first subsample included 229 offspring belonging to 100 nuclear families with DD who are part of an ongoing project on the genetic basis of DD [18]. The second subsample consisted of 83 unrelated typical readers from a community-based cohort [51]. Either blood or mouthwash samples were obtained from both subsamples for DNA collection. Of the total sample (*n* = 312), a DNA sample was available from 302 subjects (99 probands, 124 siblings and 79 typical readers).

### 2.2. Genotypic Assessment

Twenty SNPs from 5 DD-candidate genes (i.e., *DYX1C1*, *DCDC2*, *KIAA0319*, *ROBO1* and *GRIN2B*) were genotyped in previous studies (Table 1). We selected them because they had been significantly associated with DD-related phenotypes in at least one independent sample. Exons 2 and 10 of the *DYX1C1* gene were amplified from genomic DNA (primer sequences and amplification protocols are available from the authors on request). A 0.5 microlitre aliquot of each amplified DNA sample was labelled with a BigDye Terminator Cycle Sequencing Kit (Applied Biosystems, Monza, Italy) and sequenced on an ABI Prism 3500xL Genetic Analyzer (Applied Biosystems, Monza, Italy). Sequences were aligned with Autoassembler (Applied Biosystems) and scored for known and new polymorphisms. Subjects were assessed for polymorphisms at rs3743205G/A, rs57809907G/T and rs189983504 C/G. Genotyping of the intron 2 deletion of READ1 was described previously [18]. Briefly, the common 2445-bp deletion was genotyped by allelic-specific amplification with a combination of three primers in one reaction. Markers *DCDC2*-rs793862A/G, *DCDC2*-rs793842C/T and *KIAA0319*-rs2038137G/T were typed by PCR amplification followed by sequencing (primer sequences are available on demand). Polymorphisms rs333491A/G, rs6803202C/T, rs9853895C/T and rs7644521T/C in *ROBO1*, rs4504469C/T and rs9461045C/T in *KIAA0319* and rs2143340A/G in *TTRAP* (covering the 77-kb region spanning the gene *TTRAP* and the first four exons of the neighboring gene *KIAA0319* and found to be associated with DD) were analyzed with quantitative PCR and typed using TaqMan SNP Genotyping assays (Life Technologies) on a 7900HT Sequence Detection System (Life Technologies). Amplifications of markers rs5796555-/A, rs1012586G/C, rs2268119A/T, rs2216128T/C, rs11609779C/T and rs2192973C/T in *GRIN2B* were performed in 10-microliter reactions using JumpStart Red ACCUTaq LA DNA polymerase (Sigma) and the following protocol: 30 s at 96 °C, 35 cycles of 15 s at 94 °C/20 s at 58 °C/30 s at 68 °C, 5 min final elongation time. Sequencing reactions were performed with a BigDye Terminator Cycle Sequencing Kit (Applied Biosystems, Monza, Italy) and ran on an ABI Prism 3500xL Genetic Analyzer (Applied Biosystems, Monza, Italy) (primer sequences available upon request). Table 1 shows allelic frequencies and Hardy-Weinberg equilibrium (HWE) for the selected markers calculated in the unrelated subjects (i.e., probands with DD and typical readers). Genotype distributions did not significantly deviate from the HWE.

The linkage disequilibrium structure of each gene was analyzed using only the unrelated subjects; linkage disequilibrium was obtained and laid out in Haploview 4.0 (Figure S1).

For those SNPs with a minor allele frequency (MAF) ≥ 35% (i.e., *DCDC2*-rs793842C/T, *KIAA0319*-rs4504469C/T, *KIAA0319*-rs2038137G/T, *ROBO1*-rs333491A/G, *ROBO1*-rs6803202T/C, *ROBO1*-rs9853895C/T), the additive genetic model was tested and the genotypes were classified into three-level variables. For all the other SNPs, the effect of the presence/absence of the minor allele was tested and the genotypes were classified into two-level variables.

### 2.3. Endophenotypic Assessment

#### 2.3.1. Rapid Auditory Processing: Temporal Order Judgment Task

RAP was assessed by a temporal order judgment task using two complex tones composed of frequencies within the speech range, each lasting 40 ms. The two tones differed in their fundamental frequency (A: F0 = 100 Hz for the low tone and B: F0 = 305 Hz for the high). Stimulus pairs were created by placing the two stimuli into the two possible combinations (AB and BA; chance level = 50%) with five randomly presented different inter-stimulus intervals (ISIs; i.e., 20, 40, 80, 120 and 280 ms). The children had to indicate the order of the tones after each trial, while the experimenter entered their responses pressing the corresponding key on the computer keyboard; no visual feedback on response accuracy was provided. Each trial started with the appearance of the fixation point (500 ms), and the participants were instructed to keep their eyes on it throughout the trial. The experimental session consisted of 40 trials (8 trials × 5 ISIs). The dependent variable was the mean among percentage of response accuracy for each ISI. An eight-stimulus pair with an ISI of 500 ms training session was held to familiarize the children with the task; visual feedback on response accuracy was provided. A value was then conferred to each ISI for each participant (i.e., 1 for “below or equal to the 25th percentile of distribution”; 2 for “between the 25th and the 75th percentile of distribution”; 3 for “above or equal to the 75th percentile of distribution”) according to the distribution obtained from the total sample.

#### 2.3.2. Rapid Automatized Naming

Cross-modal mapping from visual stimuli to the correspondent spoken words was measured by using a discrete rapid automatized naming task, in which a single solidly colored circle was presented (i.e., red, blue, white or green). A non-alphanumeric RAN task was used, since previous findings have shown that it predicts later reading performance [43] without being biased by reading experience or early differences in reading ability. Each trial started with the appearance of the fixation point (500 ms) and the participants were instructed to keep their eyes on the fixation point (i.e., a 1° of visual angle cross appearing at the center of the screen) throughout the trial. After a blank of 50 ms, a colored circle (diameter = 4.5 cm) appeared in the center of the screen and remained there until the participant responded. The participants had to name the colors of the circles as fast as possible. The experimenter entered response accuracy by pressing the corresponding key on the computer keyboard; no feedback was provided. Both vocal RTs and error rates were recorded by the computer. The inter-trial interval was 1550 ms. The experimental session consisted of 32 discrete trials divided into two blocks of 16 trials each (4 trials for each color). The dependent variable was the mean time in milliseconds (ms, RAN_rt) for all the correctly named trials. RTs longer than 1000 ms were defined as outliers and were excluded from the data set before the analyses were carried out. In order to avoid a scaling effect in mediation analyses [70], RAN_rt was normalized within the sample.

#### 2.3.3. Multisensory Non-Spatial Attention: Visual and Auditory Attention Tasks

The description has been reported in detail in another study [71]. In the visual orienting attention task, two circles were presented peripherally, one to the left and one to the right of the fixation point. The peripheral cue involved one of the circles flashing on (40 ms in duration) and then off. The visual target stimulus (40 ms in duration) was a dot (0.5°) in the center of one of the two circles. Stimuli were white on a black background and had a luminance of 24 cd/m^2^. In the auditory orienting attention task, the sounds were transmitted through headphones. An auditory cue (40 ms in duration) consisting of a single pure tone of 1000 Hz was transmitted to either the left or the right ear followed by a target sound (40 ms in duration) consisting of a single pure tone of 800 Hz played either in the same or in the opposite ear. Each trial started with the appearance of the fixation point (i.e., a 1° of visual angle cross appearing at the center of the screen) and the participants were instructed to keep their eyes on it throughout the trial. The two lateral circles appeared on the display only in the visual orienting attention task. The cue was presented either on the right or the left after 500 ms (i.e., one of the two lateral circles for the visual task or one of the two ears for the auditory task). The cue was followed by the target at one of two cue-target stimulus onset asynchronies (SOAs; 100 or 250 ms). In response trials, the probability that the target would appear in the cued location (valid trial) or in the other location (invalid trial) was 50% (cue location was non-predictive of target location). In contrast, the target was not presented in catch trails and the participants did not have to respond. Catch trials were intermingled with response trials. The participants had to react as quickly as possible to the presence of the visual and the auditory targets by pressing the spacebar on the computer keyboard (i.e., detection task measuring simple reaction times). Both reaction times (RTs) and error rates were recorded by the computer. The maximum time allowed to respond was 1500 ms. The inter-trial interval was 1000 ms. The experimental session consisted of 160 trials divided into two blocks of 80 trials each. Trials were distributed as follows: 32 valid trials (i.e., the target appeared at the cued location; 16 for each SOA), 32 invalid trials (i.e., the target appeared at the uncued location; 16 for each SOA), and 16 catch trials (20% of total trials). The administration sequence of the two attention tasks (visual and auditory) was counterbalanced across subjects. Errors in both the visual and the auditory attention tasks were less than 3% and were not analyzed. RTs faster than 150 ms or more than 1500 ms were defined as outliers and were excluded from the data before the analyses were carried out. A mean composite score between mean correct detection RTs in both the valid and invalid trails at each SOA in the visual and in the auditory attention tasks was created. To measure the warning effect (WE), the difference between the RTs of the multisensory mean correct detection at 250 ms SOA versus 100 ms SOA was calculated [71].

#### 2.3.4. Visual Motion Processing: The Rotating-Tilted-Lines Illusion—RTLI

The description has been reported in detail in another study [59]. Briefly, the stimuli consisted of videos where the RTLI continuously contracted and expanded, varying in diameter from 12.7° to 14.6° with a speed of 5.33 mm/s, at a given contrast. Eleven Michelson contrast values were used (with a 1% step between the), ranging from 0% to 10% between RTLI and the background. Before the experiment started, the subject was familiarized with a 98% contrast RTLI and with an isoluminant colored version, by watching the patterns contract and expand on the screen. During the experiment, two tasks in the presence of the same stimuli (i.e., a detection task and an illusory effect task) were performed by the participants. In each detection task trial, the participants had to report whether the circle of lines was present or not. The aim was to obtain a contrast detection threshold under the same conditions as the illusory effect task. In each illusory effect task trial, the subjects had to report whether rotation was perceived or not. The participants viewed the stimuli binocularly without time constraints. Each video was presented five times in random order. The individual curves, representing performance in the illusory effect task, were fitted by a logistic function. The upper bound was set at 1, and the lower bound at y_0_ = 0, where y = 0 means that the illusory rotation was never perceived, and y = 1 that it was always perceived. The free parameters of the function b (the function slope; RTLI_b) and t (the 50% threshold; RTLI_t) were submitted to the analyses. The resulting logistic function is as follows:

y = 1/1 + e^−b(x−t)^ (1)

where x represents the percentage of contrast increment between the RTLI and the background and y the correlated response frequency.

Mediation analyses required that variables should be approximately normally distributed [70]. We therefore transformed RTLI_b via logarithm transformation and RTLI_t via square root transformation before running analyses, to obtain acceptable distributions [46].

### 2.4. Outcome Assessment

Reading outcome was assessed by text [72], single unrelated words and pseudo-words [73] reading tests. The text-reading task evaluated the ability to read meaningful material increasing in complexity according to grade level, and provided separate scores for speed and accuracy. Norms were provided for each text [72]. The single words and pseudo-words reading tasks assessed speed and accuracy (number of errors) in reading word (four lists of 24 words) and psuedo-word lists (three lists of 16 pseudo-words), and provided grade-level norms from the second to the eighth grades [73]. Mean bivariate correlations (*r*) were substantial (*r* = 0.548; data available upon request); therefore, we created a reading composite score. Table S1 shows the descriptive statistics of all study variables for the whole sample.

### 2.5. Statistical Analysis

Direct correlations between gene and EPs, gene and reading, and EPs and reading, were calculated using two-tailed bivariate Pearson correlations as implemented in IBM SPSS Statistics for Windows, Version 21.0 (IBM Corp. Released, 2012).

**Figure 1:**
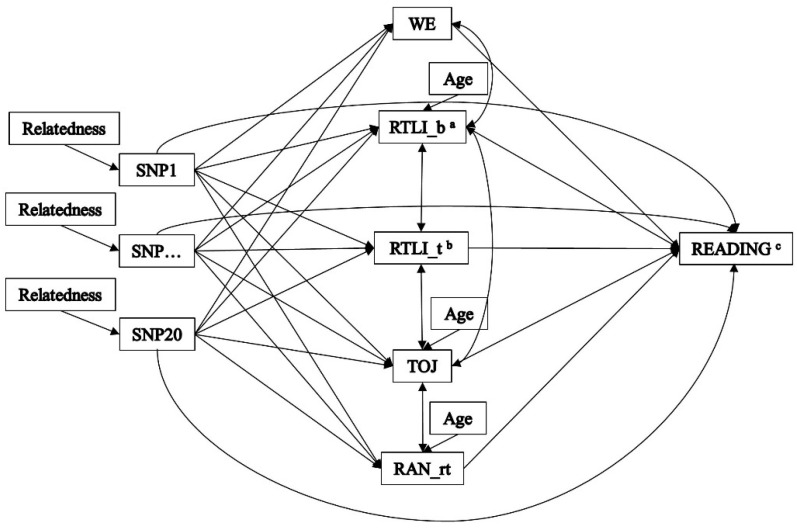
The multiple-predictor/multiple-mediator model. WE = multisensorial warning effect; RTLI_b = rotating-tilted-lines illusion, slope; RTLI_t = rotating-tilted-lines illusion, threshold; RAP = rapid auditory processing; RAN_rt = rapid automatized naming of colors, reaction time. Family relatedness was controlled as clustering variable upon the SNPs’ effects. We also controlled for the correlations between RTLI_b, RAP and RAN_rt, and “age”, between between WE and RTLI_b, between RAP and RTLI_b, RTLI_t and RAN_rt, and between RTLI_b and RTLI_t (Table S2). ^a^ It refers to values after logarithm transformation. ^b^ It refers to values after square root transformation. ^c^ It refers to the average among text-, single word and single non-words reading tasks (both accuracy and speed).

Indirect effects were tested by a multiple-predictor/multiple-mediator model using Structured Equation Modelling (SEM) as implemented in the MPlus software package (Figure 1) [74]. SEM concurrently models all paths, giving more powerful, accurate and robust estimation of mediation effects than more traditional tests based on sequential regressions, especially when more than one mediator is implemented in the model. All of the relationships among variables in the model are tested together and all paths can be compared with each other in terms of each variable’s degree of importance [70]. Indirect effects were examined using the 5000 bootstrap technique to assess non-normality in the product coefficient [75]. Confidence intervals (95% CIs) that did not contain zero indicated significant indirect effects [76]. This method offers the best power, confidence interval placement, and overall control for Type I error [70]. As no golden rule exists to assess model fit, reporting a variety of indexes is recommended to reflect different aspects of model fit [70]. The goodness-of-fit of the model was therefore evaluated by use of the chi-square statistic, the standardized root mean square residual (SRMR, with values ≤ 0.08 indicating adequate fit), the root mean square error of approximation (RMSEA, with values ≤ 0.08 indicating adequate fit), and the comparative fit index (CFI, with values ≥ 0.95 indicating adequate fit).

As part of the sample consisted of siblings, to control for the degree of kinship, we considered relatedness (i.e., proband versus sibling) as a clustering variable upon the SNPs’ effects. Moreover, as “age” was significantly correlated with RTLI_b, RAP and RAN_rt (Table S2), we controlled these measures for the effect of age. Finally, as collinearity plays a role in multiple mediation models as it does in ordinary multiple regression [65], we controlled for the correlations between WE and RTLI_b, between RAP and RTLI_b, RTLI_t and RAN_rt, and between RTLI_b and RTLI_t (Table S2).

## 3. Results

### 3.1. Bivariate Associations between Gene and EPs, Gene and Reading, and EPs and Reading

#### 3.1.1. Bivariate Associations between Gene and EPs

The *DYX1C1*-rs3743205, *DYX1C1*-rs57809907 and *DYX1C1*-rs189983504 SNPs significantly correlated with RTLI_b, RAP, and RAN_rt, respectively; the *ROBO1*-rs333491 and *ROBO1*-rs9853895 SNPs significantly correlated with both RTLI_t and RAN_rt, and the *ROBO1*-rs6803202 SNP significantly correlated with WE; the *GRIN2B*-rs2216128 and *GRIN2B*-rs2192973 significantly correlated with RAP (Table 2).

#### 3.1.2. Bivariate Associations between Gene and Reading

Significant correlations were found between the *DCDC2*-rs793842, *ROBO1*-rs333491, *ROBO1*-rs9853895 and reading (Table 2).

#### 3.1.3. Bivariate Associations between EPs and Reading

All EPs revealed a significant association with reading (WE: *r* = −0.160, *p* = 0.005; RTLI_b: *r* = 0.263, *p* < 0.001; RTLI_t: *r* = −0.144, *p* = 0.031; RAP: *r* = 0.329, *p* < 0.001; RAN_rt: *r* = −0.252, *p* < 0.001; Table S2).

### 3.2. Indirect Effects—The Multiple-Predictor/Multiple-Mediator Model

The multiple-predictor/multiple-mediator model provided a good fit to the data (χ^2^_(9)_ = 26.212, *p* = 0.001; RMSEA = 0.087, 90% CI = 0.051–0.125, CFI = 0.950; SRMR = 0.014) and explained 33.9% of the variance in reading skills. Post-hoc power calculation for the multiple-predictor/multiple-mediator model was conducted using the R code by Quantpsy (http://quantpsy.org/rmsea/rmsea.htm) to compute power for RMSEA with alpha set at 0.05. The analysis was modelled for 8 degrees of freedom, sample size of 302 subjects and RMSEA = 0.087. Under these assumptions, the estimated statistical power was above 80%.

**Figure 2:**
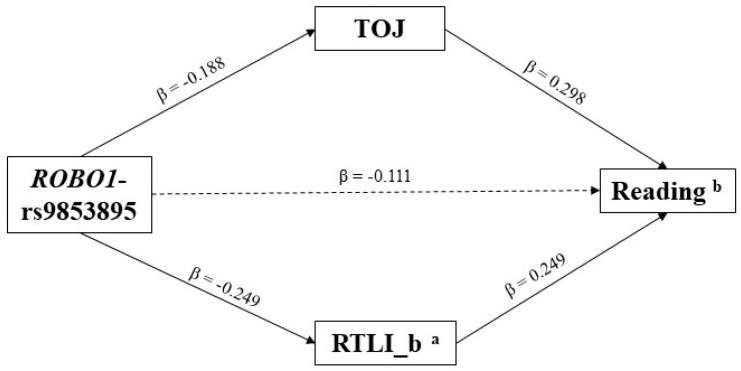
Significant specific indirect effects within the significant total indirect effect from ROBO1-rs9853895 to Reading (standardized estimates of path coefficients are depicted). RAP = Rapid auditory processing; RTLI_b = Rotating-Tilted-Lines Illusion, slope. Family relatedness was controlled as clustering variable upon the SNPs’ effects (cf. “2.5 Statistical Analysis” paragraph). The effect of age was controlled as covariate upon RAP and RTLI_b (cf. “2.5 Statistical Analysis” paragraph). Non-significant paths are indicated by a dotted line. ^a^ It refers to values after logarithm transformation. ^b^ It refers to the average among text-, single word and single non-words reading tasks (both accuracy and speed).

Using 5000 bootstrapping analyses and bias-corrected 95% CI, we found a significant total indirect effect from *ROBO1*-rs9853895 to reading (Table 3). Within this pathway, two specific indirect effects were significant, involving RAP and RTLI_b as mediators (Table 4). Inspection of beta scores revealed that the specific indirect effect along both pathways was negative. Specifically, the T/T genotype group predicted impairments in RAP and visual motion processing, which, in turn, predicted poorer reading skills (Figure 2). Post-hoc power calculations for the specific indirect effects were conducted using the computer software MedPower (https://davidakenny.shinyapps.io/PowerMed/) to estimate power for a given sample size with alpha set at 0.05. The analysis was modelled for (i) gene→EP paths of −0.188 and −0.249, respectively; (ii) EP→reading paths of 0.298 and 0.249, respectively; and (iii) for gene→reading path of −0.111. Under these assumptions, the estimated statistical power of both the specific indirect effects was above 90%.

## 4. Discussion

Building on previous results demonstrating solid cognitive and sensory EPs of DD [51], this study simultaneously examined the presence of the direct effects of 20 SNPs spanning five DD-candidate genes on reading skills, as well as indirect pathways involving performance on EPs as mediators of these associations, using a multiple-predictor/multiple-mediator framework. According to our hypotheses, indirect effects were accounted for by the *ROBO1*-rs9853895C/T SNP on RAP and visual motion processing, and explained about 40% of the variance in reading skills. As hypothesized by the partial mediational model [20,25], these findings suggested that part of the genetic effect on the phenotype is mediated through EPs. Consistent with the multiple deficits model underlying the liability of complex traits [77,78], the direct effect of genetic variation is limited and represents only the first step in a sequence of events that may ultimately lead to the behavioral phenotype [21]. Therefore, testing EPs as mediating variables may be an effective approach to arriving at a clearer understanding of the relationship between the genetic and cognitive underpinnings of symptoms of behavior [21,70].

The current findings support our previous results, implicating RAP and visual motion processing as the most solid EPs of DD [51], and provide further support for the role of deficits in the processing of transient and dynamic auditory and visual stimuli in the etiology of DD [27,28,36,37,42,47,48,79,80,81,82,83,84,85,86,87,88]. These findings further support the dominant, albeit controversial account [82,83], of the M-D theory of DD [47]. According to the general M-D theory [35,47,80,83,84,86], DD is due to a multimodal sensory impairment in the processing of transient and dynamic stimuli [36,83,85,86], which might arise from a deficit in neural pathways involved in the fast transmission and processing of sensory information [82,84,89]. Successful sequencing depends on the accurate timing of auditory and visual sensory inputs, which leads to hearing accurately the changes in the amplitude and/or frequency of the sounds and to rapidly recognizing and sequencing written letters [79]. Therefore, accurate timing facilitates the formation of precise memory representations of the order of sounds (phonological processing) and letters in a word (orthographic processing). This ability depends upon deploying attention accurately and in the correct sequence [33,36,89]. Such sequential allocation of attention depends upon the properties of “transient” systems in the brain, which is mediated by networks of “magnocellular” neurons whose size enables them to react rapidly to temporal transients [36,50,79,90,91,92,93]. Segregated magno- and parvo-cellular processing routes are well documented in the visual system from the lateral geniculate nucleus up to the level of the primary visual cortex [94,95,96]. Although similar magno/parvo distinction is not typically made in the auditory system, magno cells also exist in the medial geniculate nuclei. Additionally, auditory analogies to magno- and parvo-cellular auditory processing streams have been suggested at the cortical level [97,98,99,100,101]. These multi-sensory deficits in dynamic processing of transient stimuli [79] could be linked with typical impairments in integrating visual symbols with their corresponding speech sounds. Although there is a debate about causal relationships between multisensory dynamic processing and print-to-speech sound integration, as well as their neural bases, these processes all require precise and rapid timing mechanisms across distributed brain networks in which perceptual neural noise exclusion is fundamental [79,102,103,104,105,106].

Furthermore, our findings are consistent with recent evidence showing that *ROBO1* affects the development of the central nervous system during the embryonic and fetal stages [107,108], and of the sensory pathways involved in the reading acquisition process [108]. *ROBO1* encodes a receptor protein for the SLIT family of proteins, and plays an essential role in axon guidance (e.g., midline crossing and neuronal migration of precursor cells) [107,109,110,111,112,113,114,115], as demonstrated by both RNAi and knockout experiments in mice and rats [107,116,117,118,119,120]. Thus, the present study builds upon these past works by corroborating indirect pathways linking variants spanning *ROBO1* with reading (dis)ability via *ROBO1*′s effects upon rapid auditory and visual motion processing. Our data support the hypothesis that *ROBO1* may influence changes in brain systems underlying these cognitive EPs of reading. These results agree with recent studies that have examined connections among cognitive processes, genetics and behavior in learning skills [121,122,123,124].

There are limitations to the current study. First, although a comprehensive battery of cognitive EPs was used, it would be beneficial to include additional cognitive domains, such as PA, to understand the pathophysiology of reading (dis)ability. The relationship between PA and reading has been well-established, and deficits in PA are one of the best-documented aspects of DD [125,126,127,128,129,130,131]. However, there is evidence that low-level auditory and visual sensory-processing deficits come before and underlie PA deficits [36,132,133]. Second, our results are limited to decoding skills and could not be generalized to more complex reading-related traits (e.g., reading comprehension). However, it is plausible to hypothesize that an improvement in decoding speed and accuracy may have a subsequent effect on reading comprehension as it would lead to, respectively, a lower load in the working memory and to a more accurate access to the lexical meaning. Third, we cannot determine causal influences among the measures over time because of the cross-sectional nature of the study and the statistical method used. Consequently, longitudinal studies are needed to address this issue. Fourth, the markers that we selected for our study were not found to be associated with DD-related traits by GWAS [6,7,8,9] and in a large cross-linguistic sample [10]. The fact that GWAS did not confirm findings from association studies does not necessarily imply that previously reported associations were due to low statistical power and chance findings. The lack of replication of candidate genes studies may be explained by other viable reasons, such as different ethnic origin among the different samples, different linguistic environments, different inclusion criteria, gene-specific factors [10,18]. Even if the emergence of GWAS has caused a remarkable shift in our capacity to understand the genetic basis of human disease, several limitations and concerns have also been reported [134]. It is now recognized that GWAS and candidate-gene studies should be viewed as complementary rather than mutually exclusive approaches to understand complex neurodevelopmental disorders [135]. Assessing the mediating role of EPs/IPs in the pathway from genes to DD by testing the top hits from previous GWAS, should be considered for future studies. Fifth, although the sample size is smaller than classical candidate genetic studies, it is sizeable for combined gene-cognition-behavior approaches. The costs associated with such a thorough evaluation of the phenotype are an insurmountable limit to achieving the strict threshold for the GWAS statistical power. However, GWAS should not be the benchmark for power calculation when applying a deep-phenotyping candidate approach. On the contrary, it has been suggested that in the context of deep-phenotyping studies based on historical candidate genes, sample size standards should be study-specific and based on the best trade-off between data quality and sampling effort [136]. The present SEM approach yielded good estimated post-hoc statistical power for both the total indirect effect and specific indirect effects. These findings support the use of EPs for tracing effects of genetic variants on reading and for unravelling the complex pathways between a specific genetic variant and a behavioral phenotype [21,22]. Moreover, we are able to truly capture 95% of the distribution and to increase statistical power by using 95% CIs and resampling methods like the bootstrap for testing the mediated effects [137,138,139]. However, as literature on the DD-candidate genes is now large and contains a number of inconsistent findings [11], replications in independent, larger datasets are needed.

## 5. Conclusions

This first-time investigation of the etiological sequence from 20 SNPs spanning five historical DD-candidate genes to reading skills via cognitive EPs contributes to the growing literature on the cognitive neurogenetic machinery of reading development. Furthermore, these findings add to a growing body of literature implicating EPs as viable and valuable markers for both genetic mapping of complex neurodevelopmental disorders and, potentially, helping reshape classical nosological systems and diagnostic categories [20,21]. Finally, by showing potential sequential effects, whereby variants in DD-candidate genes drive functioning in cognitive EPs that contribute to reading outcome, this study paves the way for new potential interventions. Specifically, treatments that target deficits in specific EPs [20] are likely to be more effective for some groups of children and the degree of response to such interventions may be partially regulated by genetic factors. As treatments focused on RAP and visual motion processing have been shown to improve reading skills in children with DD [33,36,140,141,142,143,144,145], our results suggest they may be especially warranted in carriers of *ROBO1*′s risk allele, hopefully with enduring educational, psychosocial and economic repercussions.

## Figures and Tables

**Table 1:** Allele frequencies and Hardy-Weinberg equilibrium’s *p*-values.

		Allele	Frequency in Unrelated Subjects *	Hardy-Weinberg Equilibrium
*DYX1C1*	rs3743205	G	0.927	0.018
		A	0.073	
	rs57809907	G	0.891	0.984
		T	0.109	
	rs189983504	C	0.899	0.546
		G	0.101	
*DCDC2*	rs793842	C	0.584	0.196
		T	0.416	
	READ1	Deletion °	0.078	0.286
	rs793862	G	0.758	0.581
		A	0.242	
*KIAA0319*	rs4504469	C	0.634	0.527
		T	0.366	
	rs2038137	G	0.642	0.556
		T	0.358	
	rs9461045	C	0.792	0.306
		T	0.208	
	rs2143340 ^§^	A	0.839	0.181
		G	0.161	
*ROBO1*	rs333491	A	0.545	0.378
		G	0.455	
	rs6803202	C	0.494	0.361
		T	0.506	
	rs9853895	C	0.587	0.232
		T	0.413	
	rs7644521	T	0.836	0.744
		C	0.164	
*GRIN2B*	rs5796555	-	0.694	0.498
		A	0.306	
	rs1012586	G	0.695	0.126
		C	0.305	
	rs2268119	A	0.768	0.729
		T	0.232	
	rs2216128	A	0.784	0.593
		G	0.216	
	rs11609779	C	0.817	0.310
		T	0.183	
	rs2192973	G	0.775	0.410
		A	0.225	

* Probands with developmental dyslexia (DD) and typical readers. ° Microdeletion of the compound short tandem repeat in intron 2 of *DCDC2*. ^§^ Marker rs2143340A/G is located on intron 2 of the *TTRAP* gene. HWE threshold: For *DYX1C1* and *DCDC2*: *p* = 0.017 (0.05/3); for *KIAA0319* and *ROBO1*: *p* = 0.013 (0.05/4); for *GRIN2B*: *p* = 0.008 (0.05/6) (Ludwig et al., 2010; Mascheretti et al., 2015). Significant HWE *p*-values are reported in bold.

**Table 2:** Correlation among candidate genes, cognitive endophenotypes and reading composite score in the total sample (*n* = 302).

GENE	SNP	COGNITIVE ENDOPHENOTYPES					READING ^#^
		ATTENTION	VISUAL MOTION PROCESSING		RAP	RAN	
		WE	RTLI_b ^a^	RTLI_t ^b^		RAN_rt	
*DYX1C1*	rs3743205G/A	−0.012	0.147 *	−0.065	0.071	0.044	−0.007
	rs57809907G/T	0.016	0.100	−0.082	0.124 *	0.030	−0.091
	rs189983504C/G	0.044	0.113	−0.074	0.031	0.173 **	0.066
*DCDC2*	rs793842C/T	0.058	−0.019	0.001	−0.080	0.108	−0.127 *
	READ1-Deletion °	0.004	0.052	−0.057	−0.010	−0.025	0.090
	rs793862G/A	0.045	−0.048	0.032	−0.008	0.056	−0.083
*KIAA0319*	rs4504469C/T	0.032	−0.104	0.080	−0.016	0.073	−0.014
	rs2038137G/T	0.028	−0.008	−0.016	0.010	0.026	0.065
	rs9461045C/T	−0.063	−0.043	0.034	0.030	0.021	−0.071
	rs2143340A/G ^§^	−0.013	−0.022	0.029	0.061	−0.006	0.000
*ROBO1*	rs333491A/G	−0.031	−0.115	0.141 *	−0.017	−0.117 *	0.136 *
	rs6803202C/T	−0.119 *	−0.029	0.026	−0.040	−0.084	0.080
	rs9853895C/T	0.088	−0.131	0.158 *	−0.107	0.168 **	−0.195 **
	rs7644521T/C	−0.029	−0.013	0.005	0.014	0.018	0.097
*GRIN2B*	rs5796555-/A	0.030	0.018	−0.110	−0.059	0.082	−0.033
	rs1012586G/C	0.052	0.048	−0.060	−0.027	0.086	0.014
	rs2268119A/T	−0.053	−0.008	−0.055	−0.037	0.061	−0.007
	rs2216128A/G	−0.089	−0.035	0.023	−0.135 *	−0.010	−0.023
	rs11609779C/T	−0.035	0.039	−0.059	0.087	0.093	−0.069
	rs2192973G/A	−0.084	−0.055	0.071	−0.165 *	0.004	−0.068

WE = multisensorial warning effect; RTLI_b = rotating-tilted-lines illusion, slope; RTLI_t = rotating-tilted-lines lllusion, threshold; RAP = rapid auditory processing; RAN_rt = rapid automatized naming of colors, reaction time. ^a^ It refers to values after logarithm transformation. ^b^ It refers to values after square root transformation. ^#^ It refers to the average among text-, single words and single non-words reading (both accuracy and speed) as described in the text. ° Microdeletion of the compound short tandem repeat in intron 2 of *DCDC2*. ^§^ Marker rs2143340A/G is located on intron 2 of the *TTRAP* gene. * Two-tail *p* ≤ 0.05; ** two-tail *p* ≤ 0.01.

**Table 3:** Total indirect effects from single nucleotide polymorphisms (SNPs) to reading in the multiple-predictor/multiple-mediator model (standardized βs and SEs are reported).

	β	SE	95% CI *
*DYX1C1*-rs3743205	−0.004	0.039	−0.442/0.436
*DYX1C1*-rs57809907	0.055	0.038	−0.154/0.585
*DYX1C1*-rs189983504	−0.010	0.030	−0.293/0.243
*DCDC2*-rs793842	−0.015	0.035	−0.147/0.107
*DCDC2*-READ1d °	−0.020	0.031	−0.432/0.191
*DCDC2*-rs793862	0.018	0.033	−0.172/0.311
*KIAA0319*-rs4504469	−0.018	0.035	−0.142/0.100
*KIAA0319*-rs2038137	0.001	0.035	−0.127/0.118
*KIAA0319*-rs9461045	−0.046	0.050	−0.491/0.220
*KIAA0319*-rs2143340 ^§^	0.060	0.045	−0.143/0.554
*ROBO1*-rs333491	−0.018	0.029	−0.132/0.078
*ROBO1*-rs6803202	−0.057	0.038	−0.215/0.042
*ROBO1*-rs9853895	−0.099	0.042	*−0.306/−0.007*
*ROBO1*-rs7644521	0.013	0.028	−0.169/0.260
*GRIN2B*-rs5796555	−0.058	0.045	−0.497/0.127
*GRIN2B*-rs1012586	0.019	0.046	−0.275/0.356
*GRIN2B*-rs2268119	0.015	0.041	−0.248/0.331
*GRIN2B*-rs2216128	0.065	0.083	−0.492/0.881
*GRIN2B*-rs11609779	−0.002	0.030	−0.225/0.199
*GRIN2B*-rs2192973	−0.100	0.085	−0.996/0.382

* Significant coefficients are reported in italics and underlined. ° Microdeletion of the compound short tandem repeat in intron 2 of *DCDC2*. ^§^ Marker rs2143340A/G is located on intron 2 of the *TTRAP* gene.

**Table 4:** Specific indirect effects of endophenotypes (Eps) from *ROBO1*-rs9853895 to reading (standardized βs and SEs are reported).

		β	SE	95% CI *
ATTENTION	WE	−0.002	0.015	−0.062/0.048
VISUAL MOTION PROCESSING	RTLI_b °	−0.062	0.033	*−0.231/−0.006*
	RTLI_t ^§^	0.038	0.031	−0.020/0.192
RAP		−0.056	0.027	*−0.194/−0.003*
RAN	RAN_rt	−0.017	0.012	−0.076/0.007

WE = multisensorial warning effect; RTLI_b = Rotating-Tilted-Lines Illusion slope; RTLI_b = Rotating-Tilted-Lines Illusion threshold; RAP = rapid auditory processing; RAN_rt = rapid automatized naming of colors, reaction time. * Significant coefficients are reported in italics and underlined. ° It refers to values after logarithm transformation. ^§^ It refers to values after square root transformation.

## Supplementary Materials

The following are available online at https://www.mdpi.com/2076-3425/10/12/993/s1, Figure S1: Haploview plot showing pairwise linkage disequilibrium for each gene based on genotypes of unrelated subjects (i.e., probands with DD and typical readers), Table S1: Descriptive statistics of the demographic, neuropsychological and cognitive measures, Table S2: Correlation among the cognitive EPs, age and reading in the total sample (*n* = 302).
