# Supplementary material for: The Mediation Role of Dynamic Multisensory Processing Using Molecular Genetic Data in Dyslexia
Source: Brain Sci. 2020 Dec 16;10(12):993. doi: 10.3390/brainsci10120993 (PMC7765588; doi:10.3390/brainsci10120993)
Supplement: Supplementary file 1 [file brainsci-10-00993-s001.pdf]

Figure S1. Haploview plot showing pairwise linkage disequilibrium for each gene based on genotypes of unrelated subjects (i.e., probands with DD and typical readers).

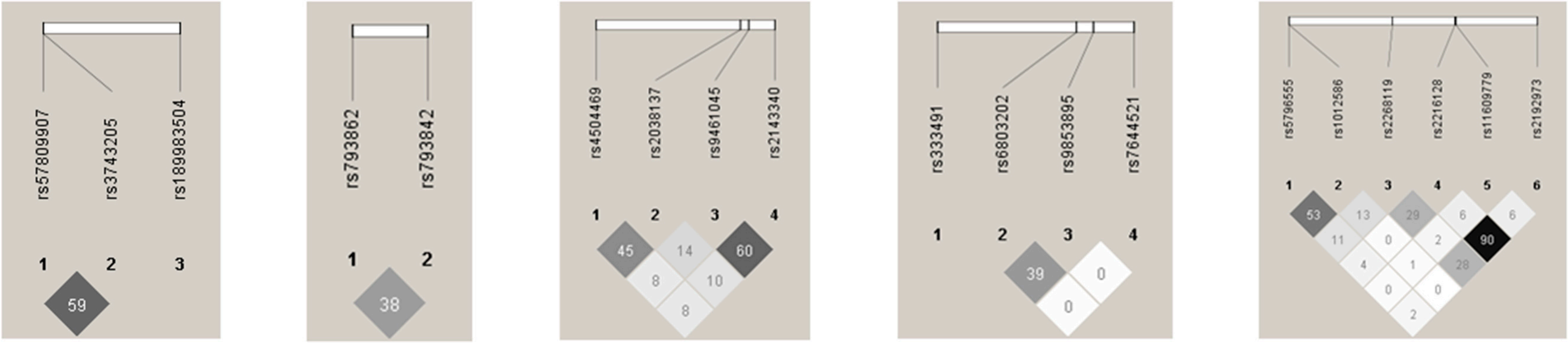

DYX1C1 ***DCDC2*** ***KIAA0319*** ***ROBO1*** ***GRIN2B***

Numbers in squares are r^2 values.

Table S1. Descriptive statistics of the demographic, neuropsychological and cognitive measures.

		Total sample (n=302) Age[a]=136.38±33.55; sex[b]=58.9%; SES[c]=59.76±19.61			Nuclear families (n=100)							General population (n=79) Age[a]=122.25±23.92; sex[b]=50.6%; SES[c]=71.07±15.30		
					All offspring (n=223) Age[a]=141.41±35.07; sex[b]=61.9%; SES[c]=55.89±19.46			Probands (n=99) Age[a]=132.19±28.09 sex[b]=67.7% SES[c]=55.20±19.37	Siblings (n=124) Age[a]=148.82±38.23 sex[b]=57.3% SES[c]=56.45±19.59	Affected (n=148) Age[a]=135.81±30.42 sex[b]=65.5% SES[c]=54.45±19.06	Unaffected (n=75) Age[a]=152.37±40.77 sex[b]=54.7% SES[c]=58.72±20.05			
		Mean±SD	Skew.	Kurtosis	Mean±SD	Skew.	Kurtosis	Mean±SD	Mean±SD	Mean±SD	Mean±SD	Mean±SD	Skew.	Kurtosis
IQ[d]	TIQ	111.14±16.81	0.20	-0.52	110.82±17.30	0.30	-0.44	111.43±16.28	110.32±18.11	109.36±16.65	113.68±18.29	110.09±15.40	-0.16	-0.87
	VIQ	105.57±17.65	0.29	-0.40	105.12±17.76	0.39	-0.37	104.64±16.73	105.49±18.57	103.37±17.02	108.49±18.77	106.83±17.38	0.02	-0.32
	PIQ	113.89±19.95	0.07	-0.15	114.01±20.54	0.10	-0.16	115.94±20.51	112.53±20.52	113.22±20.73	115.53±20.21	113.56±18.29	-0.05	-0.24
READING[e]		-0.98±1.52	-1.36	3.26	-1.42±1.52	-1.20	3.28	-2.34±1.44	-0.68±1.14	-2.13±1.34	0.00±0.55	0.26±0.44	-0.88	0.88
ATTENTION	WE	-15.90±32.97	-0.06	-0.10	-12.30±33.66	-0.21	0.00	-9.75±33.67	-14.39±33.64	-12.13±33.40	-12.63±34.37	-25.93±28.87	0.21	0.04
VISUAL MOTION PROCESSING	RTLI_b[f]	0.12±0.93	0.52	-0.96	-0.04±0.84	0.58	-0.84	-0.20±0.77	0.10±0.88	-0.16±0.80	0.21±0.88	0.75±1.01	-0.08	-1.60
	RTLI_t[g]	1.71±0.81	0.68	2.02	1.82±0.82	0.67	2.20	1.80±0.89	1.84±0.76	1.85±0.87	1.75±0.72	1.32±0.63	0.21	-0.67
RAP		2.08±0.60	-0.13	-1.13	2.00±0.62	0.08	-1.18	1.88±0.62	2.08±0.62	1.90±0.62	2.18±0.59	2.28±0.50	-0.61	-0.35
RAN	RAN_rt	610.41±107.74	0.59	0.32	609.29±111.85	0.62	0.29	621.81±103.40	599.21±117.69	623.26±109.66	581.35±111.73	613.43±96.40	0.48	0.34

TIQ=total IQ. VIQ=verbal IQ; PIQ=performance IQ; WE=multisensorial warning effect; RTLI_b= Rotating-Tilted-Lines Illusion slope; RTLI_t=Rotating-Tilted-Lines Illusion threshold; RAP=rapid auditory processing; RAN_rt=rapid automatized naming of colors. reaction time.

[a] Age was expressed in years.

[b] Percentage of males was reported.

[c] SES is defined by parental employment coded according to the Hollingshead nine-point scale (Hollingshead, 1975).

[d] For siblings and general population, TIQ, VIQ and PIQ represent an estimated score obtained from the 'Vocabulary' and 'Block Design' subtests as described in the text.

[e] It refers to the average among text-, single word and single non-words reading tasks (both accuracy and speed) as described in the text. Measures are in SD units relative to the age-appropriate Italian population norm.

[f] It refers to values after logarithm transformation.

[g] It refers to values after square root transformation.

Table S2. Correlation among the cognitive EPs, age and reading in the total sample (n=302).

		ATTENTION	VISUAL MOTION PROCESSING		RAP	RAN	READING°
		WE	RTLI_b[b]	RTLI_t[C]		RAN_rt	
**AGE[a]**		0.094	0.144*	-0.062	0.321**	-0.519**	0.104
**ATTENTION**	**WE**		-0.138*	0.114	0.093	-0.058	-0.160**
**VISUAL MOTION PROCESSING**	**RTLI_b[b]**			-0.675**	0.176**	-0.030	0.263**
	**RTLI_t[c]**				-0.163*	0.055	-0.144*
**RAP**						-0.258**	0.329**
**RAN**	**RAN_rt**						-0.252**

WE=multisensorial warning effect; RTLI_b= Rotating-Tilted-Lines Illusion slope; RTLI_b= Rotating-Tilted-Lines Illusion threshold; RAP=rapid auditory processing; RAN_rt=rapid automatized naming of colors, reaction time.

° It refers to the average among text-, single words and single non-words reading (both accuracy and speed) as described in the text.

[a] Age was expressed in years.

[b] It refers to values after logarithm transformation.

[c] It refers to values after square root transformation.

* Two-tail $p \leq 0.05$; ** two-tail $p \leq 0.01$.
